# GFP Transgenic Medaka (*Oryzias latipes*) under the Inducible *cyp1a* Promoter Provide a Sensitive and Convenient Biological Indicator for the Presence of TCDD and Other Persistent Organic Chemicals

**DOI:** 10.1371/journal.pone.0064334

**Published:** 2013-05-20

**Authors:** Grace Hwee Boon Ng, Zhiyuan Gong

**Affiliations:** Department of Biological Sciences, NUS Graduate School for Integrative Sciences and Engineering, National University of Singapore, Singapore and Computation and Systems Biology, Singapore-MIT Alliance, Singapore, Singapore; French National Centre for Scientific Research, France

## Abstract

Persistent organic pollutants (POPs) are resistant to environmental degradation and can cause multitude of health problems. Cytochrome P450 1A (Cyp1a) is often up-regulated by POPs through the activation of aryl hydrocarbon receptor (AhR) pathway and is thus usually used as a biomarker for xenobiotics exposure. To develop a convenient *in vivo* tool to monitor xenobiotic contamination in the water, we have established GFP transgenic medaka using the inducible *cyp1a* promoter, *Tg(cyp1a:gfp)*. Here we tested *Tg(cyp1a:gfp)* medaka at three different stages, prehatching embryos, newly hatched fry and adult with 2,3,7,8-tetrachlorodiebnzo-*p*-dioxin (TCDD), a dioxin. While GFP induction was observed in all three stages, newly hatched fry were the most sensitive with the lowest observed effective concentration of 0.005 nM or 16.1 ng/L. The highly sensitive organs included the kidney, liver and intestine. With high concentrations of TCDD, several other organs such as the olfactory pit, tail fin, gills, lateral line neuromast cells and blood vessels also showed GFP expression. In addition, *Tg(cyp1a:gfp)* medaka fry also responded to two other AhR agonists, 3-methylcholanthrene and benzo[a]pyrene, for GFP induction, but no significant GFP induction was observed towards several other chemicals tested, indicating the specificity of this transgenic line. The GFP inducibility of *Tg(cyp1a:gfp)* medaka at both fry and adult stages may be useful for development of high-throughput assays as well as online water monitoring system to detect xenobiotic toxicity.

## Introduction

Polycyclic aromatic hydrocarbons (PAHs) and dioxins are persistent organic pollutant (POPs) which are resistant to environmental degradation and can persist in soils and sediments for decades or even centuries [1]. They can enter into aquatic ecosystem through effluent, atmospheric deposition, petroleum spill, run-off and ground water. With their persistent presence, POPs can be bioaccumulated and biomagnified in animal and human tissues through direct contact and food chains, thus causing health hazards such as carcinogenicity, organs failures and system dysfunction [2]–[4]. Since POPs include a variety of chemical compounds, the toxicities and affinities of POPs can be highly complicated. Currently it is widely accepted that most of the effects of POPs are mediated by aryl hydrocarbon receptor (AhR) pathway [5], which up-regulates Cyp1a enzyme that catalyzes the oxygenation of PAHs and heterocyclic aromatic amines/amids, and many other agents [6]. Cyp1a is often used as a specific and reliable biomarker for PAH contamination. As such, measurement of ethoxyresorufin-O-deethylase (EROD) activity is commonly performed to indicate the enzymatic activity of Cyp1a induced during chemical exposure [7]. Organisms that inhabit the water bodies, such as fish, are usually sacrificed to obtain their liver tissue for EROD assay.

In this study, we intended to develop a convenient biological tool to monitor PAH exposure using medaka fish (*Oryzias latipes*), which is a hardy teleost fish and is able to survive in a broad range of temperature (4°C to 40°C) as well as in a wide salinity range (0–50% of sea water) [8]. Transgenic fish have long been proposed for the purpose of biomonitoring aquatic contaminants as water sentinels [9], [10]. In these biomonitoring fish, they have been genetically modified to respond to contaminants with easily detectable reporter, based on the principle that certain genes are inducible by certain chemical contaminants. Currently, there are already a few stable transgenic fish lines established for detecting certain classes of pollutants [11]–[15]. For example, *Tg(mvtg1:gfp)* medaka, with its transgene consists of medaka vitellogenin promoter and GFP reporter gene, was established and has shown to induce GFP in the liver of male fish when exposed to estradiol [13]. Here we have developed a transgenic medaka line, *Tg(cyp1a:gfp)* in which GFP is expressed under the inducible *cyp1a* promoter, thus providing a convenient assay for easy detection *in situ* and in real time for the presence of *cyp1a*-inducing chemicals. Here we exposed the transgenic medaka to TCDD, a well studied congener of dioxin commonly used as reference point for Toxic Equivalency Factor (TEF) [2], to determine the sensitivity of the transgenic line as well as the pattern of induced GFP expression. In addition to TCDD, GFP expression in *Tg(cyp1a:gfp)* were also induced by other PAH chemicals such as 3-methylcholanthrene (3-MC) and benzo[a]pyrene (BaP), but not by other categories of chemicals such as mercury chloride, 4-nitrophenol, lindane and bisphenol A, suggesting that GFP induction in *Tg(cyp1a:gfp)* medaka is quite specific to TCDD and members of PAH family. Thus, *Tg(cyp1a:gfp)* medaka will be another convenient and useful tool in environmental monitoring.

## Materials and Methods

### Fish Husbandry

Hd-rR medaka strain was obtained from National BioResource Project, Japan [16]. Husbandry of medaka fish was according to Kinoshita et al., 2009 [8]. Staging of medaka embryos and fry was mainly based on Iwamatsu, 2004 [17]. Generation of *Tg*(*cyp1a:gfp*) [previously named *Tg(cyp1a1:gfp)*] transgenic medaka has been described previously [18]. *cyp1a* was used here because there is only one copy of *cyp1a* gene in the medaka genome [19] All experimental protocols were approved by Institutional Animal Care and Use Committee (IACUC) of National University of Singapore (Protocol 079/07).

### Toxic Chemical exposure

TCDD, 3-MC and BaP were purchased from Sigma-Aldrich. All stock solutions were prepared in dimethyl sulfoxide (DMSO) (Sigma-Aldrich) and stored at 4°C. Hemizygous transgenic embryos, fry and fish were used in all chemical exposures for consistency. Five or more fry (1–3 dph [days posthatching]) or embryos (6–9 hpf [hours postfertilization]) were placed in each well of a 6–well dish, containing 5 ml of various concentrations of a testing chemical and incubated at 28°C. Each experiment was repeated at least three times. For adult treatment, 6 month-old transgenic fish, 5 male and 5 female per tested concentration, were used and chemical solutions were changed every other day. The fish were not fed during the 72-hour chemical exposures. At the end of the experiment, all fish were examined for GFP expression. In all the acute exposure experiments, there was no mortality and no observable abnormality even with the highest concentration of TCDD (5 nM) after 24 hours of exposure.

### Epifluorescence microscopy

GFP expression was observed under an inverted fluorescence microscope (Axiovert 200 M, Zeiss) equipped with a digital camera (Axiocam HRc, ZEISS) for capturing GFP fluorescence. Fry or fish were first anaesthetized by 0.1% 2-phenoxyethanol (Sigma-Aldrich) and positioned in 3% methylcellulose (Sigma-Aldrich) for observation. For adult fish, GFP expression was observed and imaged with a stereomicroscope, Olympus MVX10 with a digital camera.

### Whole mount *in situ* hybridization

Medaka *cyp1a* cDNA was amplified from nucleotides +1321 to +1821 with reference to the transcription start site (Ensembl ID: ENSORLG00000014421) and subcloned into pGEM-T EASY (Promega, Madison, WI). Digoxigenin riboprobes of *cyp1a* was synthesized using DIG RNA labeling kit (Roche). Whole mount *in situ* hybridization was performed essentially according to the protocol described in Kinoshita et al., 2009 [15]. The period of proteinase K digestion is either 25 min to maintain surface epithelial integrity or 80 min for better penetration of riboprobes. After *in situ* hybridization, embryos were incubated with alkaline phosphatase-conjugated anti-DIG Fab Fragments (1∶7000; Roche) and were visualized with tetrazolium chloride and 5-bromo-4-chloro-3-indolyl-phosphate (Roche).

## Results

### Selection of *Tg(cyp1a:gfp)* lines for chemical tests

Four *Tg(cyp1a:gfp)* transgenic founders were initially identified and all F1transgenic progenies have been demonstrated to respond to TCDD treatment by inducing GFP expression in the liver, intestine and kidney [18]. To select a transgenic strain for monitoring xenobiotics, transgenic F1 adults that produced transgenic offspring with low or no GFP background and had a single transgenic insertion were selected, and finally a transgenic family, *Tg(cyp1a:gfp)* 4.2, with the most sensitive response to TCDD and other xenobiotic chemicals was selected. This transgenic strain, referred as *Tg(cyp1a:gfp)*, was used for all chemical exposures experiments in this study and has been maintained for five generations.

### Sensitivity and dosage-dependent GFP expression of *Tg(cyp1a:gfp)* medaka in response to TCDD

**Figure 1:**
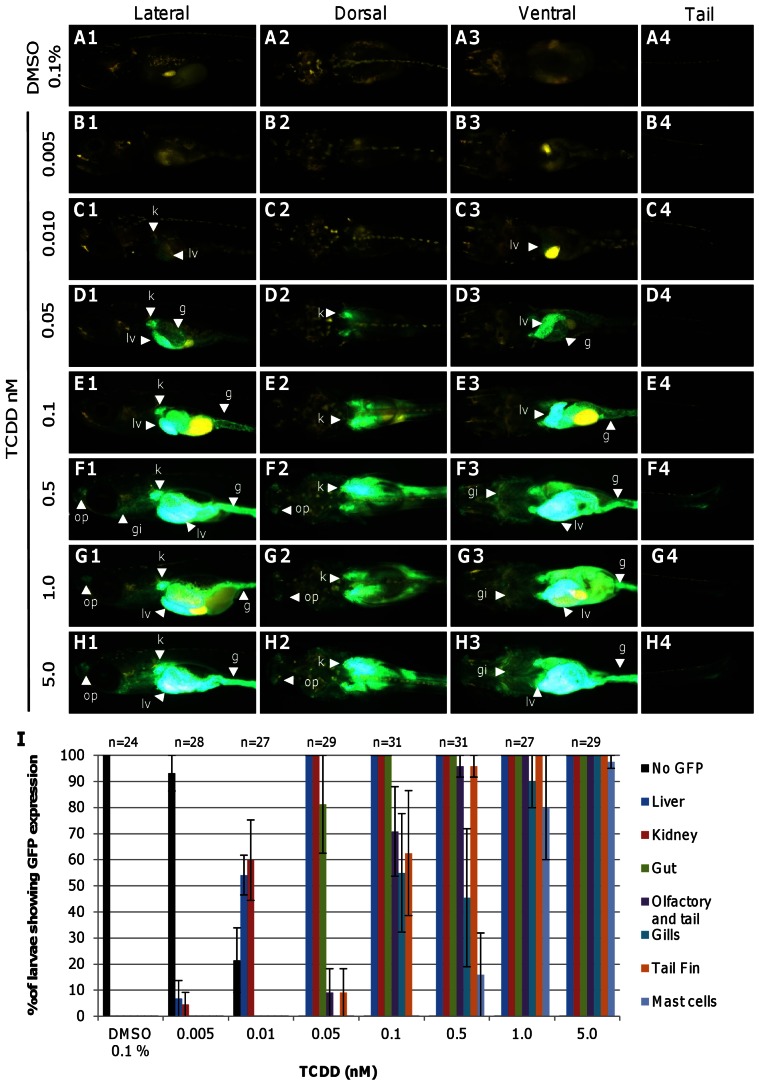
TCDD-induced GFP expression in posthatched *Tg(cyp1a:gfp)* fry. (A–H) GFP expression in *Tg(cyp1a:gfp)* fry at different concentrations of TCDD. 1–3 dph *Tg(cyp1a:gfp)* fry were exposed to TCDD for 24 hours and photographed for GFP expression under a fluorescence microscope. Lateral, dorsal, ventral and tail images were taken from the same representative fry from each concentration group. GFP expressing tissues are pointed by arrowheads. Abbreviations: g, gut; gi, gills; k, kidney; lv, liver; op, olfactory pit. (I) Percentage of fry expressing GFP in specific organs at different concentrations of TCDD. The scoring organs were based on direct observation GFP fluorescence under a fluorescence microscope. Error bars represent standard error. n represents the total number of fry used.

**Figure 2:**
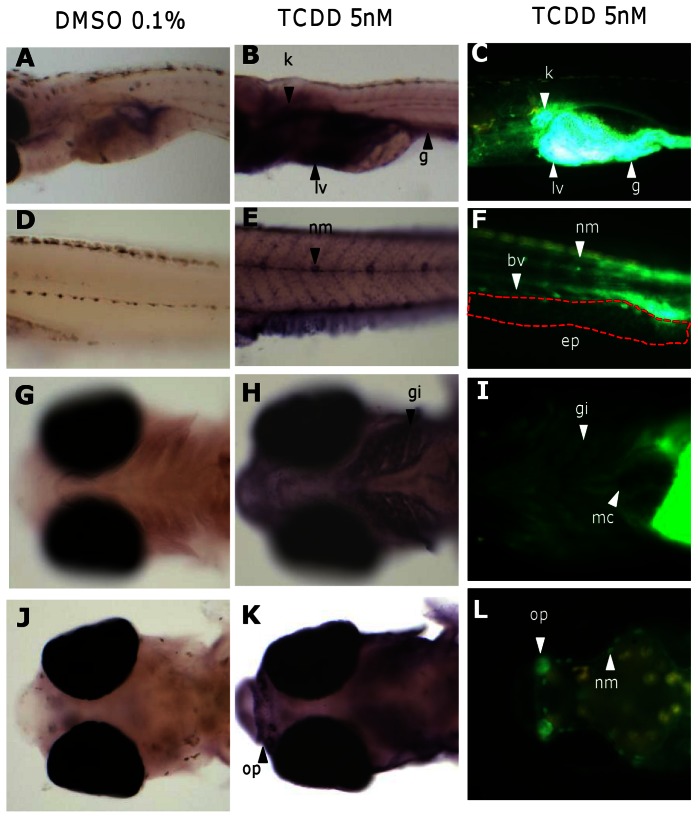
GFP induction is similar to induced endogenous *cyp1a* transcript in TCDD treatment. (A, B, D, E, G, H, J, K) Whole mount i*n situ* hybridization detection of *cyp1a* mRNAs in 0.1% DMSO-treated wild type fry (A, D, G, J) and 5 nM TCDD-treated wild type fry (B, E, H, K). (C, F, I, L) GFP expression in 5 nM TCDD treated transgenic fry. Interesting organs are indicated by arrowheads. Abbreviations: bv, blood vessel; ep, epithelia; g, gut; gi, gills; k, kidney; lv, liver; mc, myocardium; nm, neuromast cells; op, olfactory pit.

1–3 dph *Tg(cyp1a:gfp)* medaka fry were exposed to a range of concentrations of TCDD for 24 hours to determine its sensitivity to TCDD (Figure 1). No GFP expression was observed in the vehicle solvent, 0.1% DMSO-treated transgenic fry (Figure 1). About 7% of the transgenic fry responded to 0.005 nM TCDD with GFP expression in the liver (Figure 1I), indicating the lowest observed effective concentration (LOEC) was around 0.005 nM TCDD. With the increasing concentrations of TCDD, induced GFP fluorescence became increasingly intensive and appeared in more tissues/organs. For examples, at 0.05 nM, GFP expression was observed in the kidney, liver and also gut (Figure 1D) and the GFP signal became intensified in these three organs at 0.1 nM (Figure 1E). At 0.5 nM or above, GFP expression could be observed in several other organs such as olfactory pits, caudal fin, gills, neuromast cells along the trunk and around the eyes in majority of the treated transgenic fry (Figure 1F–H, also see Figure 2F,I,L). Some fry also exhibited weak GFP expression in blood vessels along the trunk (Figure 2F), myocardium and skin epithelial cells (Figure 2F,I). Quantitative GFP expression data in these major expressing organs are summarized in Figure 1I. The most sensitive organs were the kidney and liver, followed by the gut (Figure 1I).

### Induced GFP expression is consistent with induced endogenous *cyp1a* expression

To determine the endogenous *cyp1a* mRNA expression after TCDD exposure, we performed whole mount *in situ* hybridization with a *cyp1a* antisense probe in TCDD-treated wild type fry. The hybridization signal was observed in major internal organs including the kidney, liver and gut (Figure 2B), similar to the typical GFP expression pattern induced by TCDD in *Tg(cyp1a:gfp)* fry (Figure 2C)*;* in comparison, there was no hybridization signal detected in these organs in the vehicle control (Figure 2A). Similarly, whole mount *in situ* hybridization with sense riboprobes did not have any staining (Figure S1), thus demonstrating the specificity of the *cyp1a* antisense probes. Under a short proteinase K digestion treatment where the integrity of the epithelial surface of *in situ* hybridization sample was maintained, hybridization signals were observed in the neuromast cells along the lateral line, skin, gills and olfactory pits in TCDD-treated fry (Figure 2E,H,K), but not in the vehicle control (Figure 2D,G,J). These corresponded to the sites of GFP expression observed in 5.0 nM TCDD treated transgenic fry (Figure 2F, I, L). Overall, these observations demonstrated that induced GFP expression in *Tg(cyp1a:gfp*) embryos/fry faithfully represent the induced endogenous *cyp1a* expression by TCDD treatment at least in these major expressing organs.

### Specificity of *Tg(cyp1a:gfp)* in response to other types of chemicals

**Figure 3:**
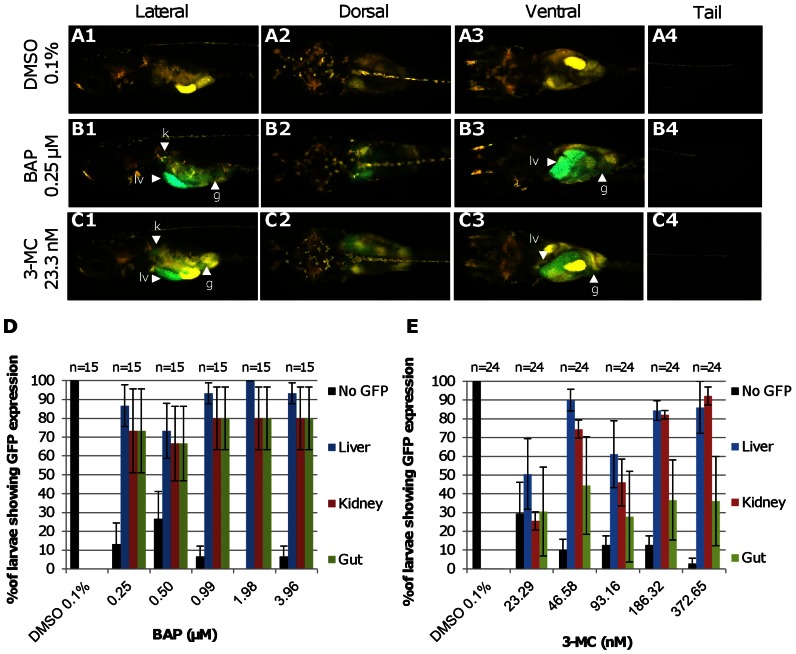
GFP induction of *Tg(cyp1a:gfp)* fry by BAP and 3-MC. (A–C) GFP expression in *Tg(cyp1a:gfp)* fry in vehicle control 0.1% DMSO (a), 0.25 µM BAP (B) and 23.3 nM 3-MC (C). GFP expressing tissues are pointed by arrowheads. Abbreviations: g, gut; k, kidney; lv, liver. (D, E) Percentages of fry expressing GFP in specific organs induced by BAP (D) or by 3-MC (E). Error bars represent standard error. n represents the total number of fry used.

To investigate the specificity of *Tg(cyp1a:gfp)* in response to other types of chemicals, transgenic fry were also exposed to other PAHs including 3-MC and BaP. Induced GFP fluorescence was observed in the kidney, liver and gut by BaP treatment (Figure 3B), in comparison with the lack of GFP expression in the vehicle control (Figure 3A). The percentages of fry expressing GFP in these three organs were quite similar in each concentration of BAP (Figure 3D) [0.25 µM (62.5 µg/l) to 3.96 µM (1000 µg/l)]. In 3-MC treatment, although GFP expression was also observed in the kidney, liver and gut (Figure 3C), the liver seemed to be the most sensitive organ, followed closely by kidney. At the lowest dosage, 23.3 nM (6.25 µg/l), ∼50% of the fry had induced GFP expression in the liver while ∼30% of the fry showed GFP expression in the gut and kidney (Figure 3E). The percentages of fry expressing GFP in the kidney and liver were higher than those in the gut for higher concentrations of 3-MC (Figure 3D) [46.6 nM (12.5 µg/l) to 372.7 nM (100 µg/l)]. Unlike TCDD exposure, GFP expression was not observed in other organs beyond the liver, kidney and gut for both 3-MC and BAP treatments. We also tested several other types of chemicals, including mercury chloride, bisphenol A (BPA), 4-nitrophenol and lindane, but there was no induced GFP expression observed in the transgenic fry, except that a small number of fry (∼20%) showed weak spotty GFP expression in the liver at the high concentration, 53.9 µM and 71.89 µM of 4-nitrophenol (Figure S2). Table 1 summarizes the information of responses of *Tg(cyp1a:gfp)* to all these chemicals, including TCDD.

**Table 1:** Summary of GFP expression induced in Tg(*cyp1a:gfp*) fry with different chemical exposures.

Chemicals	GFP expression	Lowest effective concentration
TCDD	liver, kidney, gut, olfactory pits, gills, blood vessels, neuromast cells, skin, caudal fin, myocardium	0.005–0.01 nM
3-MC	liver, kidney and gut	≤23.3 nM
BaP	liver, kidney and gut	≤247.7 nM
4-nitrophenol	Spotty GFP expression in liver	35.9–53.9 µM
Bisphenol A	No GFP induction	Nil
Mercury chloride	No GFP induction	Nil
Lindane	No GFP induction	Nil

### Induction of GFP expression in *Tg(cyp1a:gfp)* embryos

**Figure 4:**
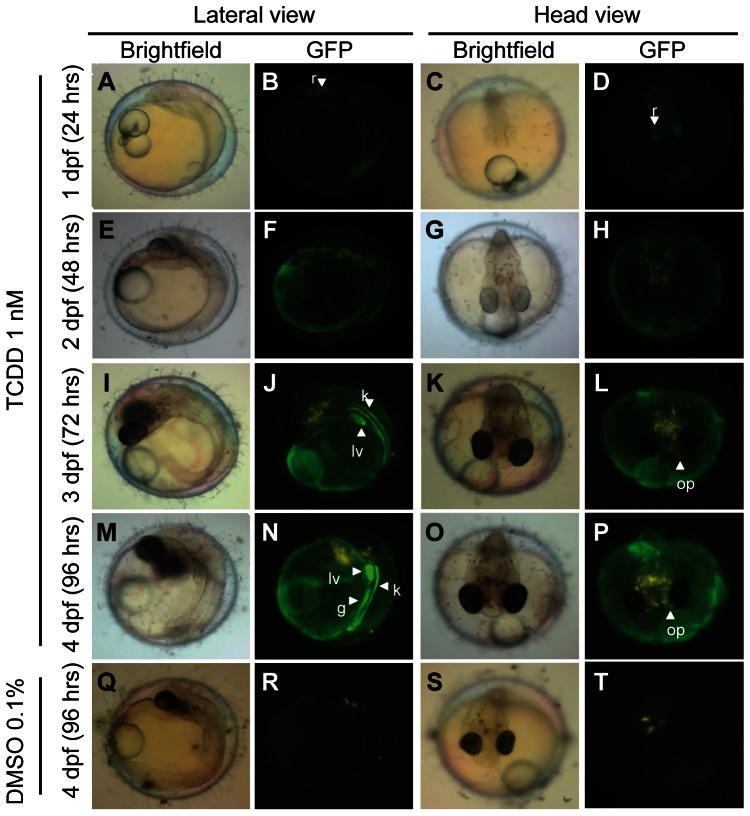
GFP induction in *Tg(cyp1a:gfp)* embryos by TCDD. 6–9 hpf hemizygous embryos were treated with 1 nM of TCDD or vehicle solvent control (0.1% DMSO) continuously. Bright field and fluorescence images of lateral and head views were taken from a representative embryo at different time points posttreatment: 24 hrs (A–D), 48 hrs (E–H), 72 hrs (I–L) and 96 hrs (M–P). No induced GFP expression was observed in the control group during the course of 96 hrs of treatment (Q–T). Interesting organs are indicated by arrowheads. Abbreviations: g, gut; k, kidney; lv, liver;op, olfactory pit.

To fully characterize the induction of GFP expression in early *Tg(cyp1a:gfp)* embryos, hemizygous transgenic embryos were exposed to TCDD continuously from ∼6 hpf (stage 10 to stage 11). It was apparent that *Tg(cyp1a:gfp)* embryos were less sensitive than post-hatching fry in TCDD induction of GFP expression. Up to 0.05 nM, there was no robust GFP fluorescence induced up to 72 hours of exposure. At 1.0 nM of TCDD, GFP fluorescence was observed weakly in the epithelial layer of yolk and embryonic body at 24 hours of exposure (Figure 4A,B). In a few embryos (6/21), moderate GFP signal was observed in the retina after 24 hours of exposure (Figure 4B,D) but diminished from 2 dpf (Figure 4F,H). At 2 dpf (∼48 hours of exposure), weak GFP expression persisted in the epithelial layer of embryonic body and the yolk (Figure 4E,F). By 3 dpf (∼72 hours of exposure), GFP was clearly expressed in the liver, kidney tubule, gut and olfactory pits (Figure 4I–L) in all TCDD- treated transgenic embryos. Longer exposure period gave similar observations to that of 3-dpf embryos (Figure 4M–P). In comparison, no GFP expression, except for a transient, faint GFP signal in the mid-brain at 1 dpf (Figure S3), was observed in the vehicle solvent group at any time of TCDD treatment (Figure 4Q–T). In summary, weak induction of GFP expression can be performed in epithelia and yolk within 24 hours of fertilization, but robust induction of GFP expression occurs only after the liver is developed at 3 dpf. These observations may indicate the possible presence of AhR activity in early stages of embryonic development. However, the pre-hatching embryos are relatively insensitive to TCDD induction compared to post-hatching fry, hinting a lower AhR activity in embryos than that in fry.

### Induction of GFP expression in *Tg(cyp1a:gfp)* adults

**Figure 5:**
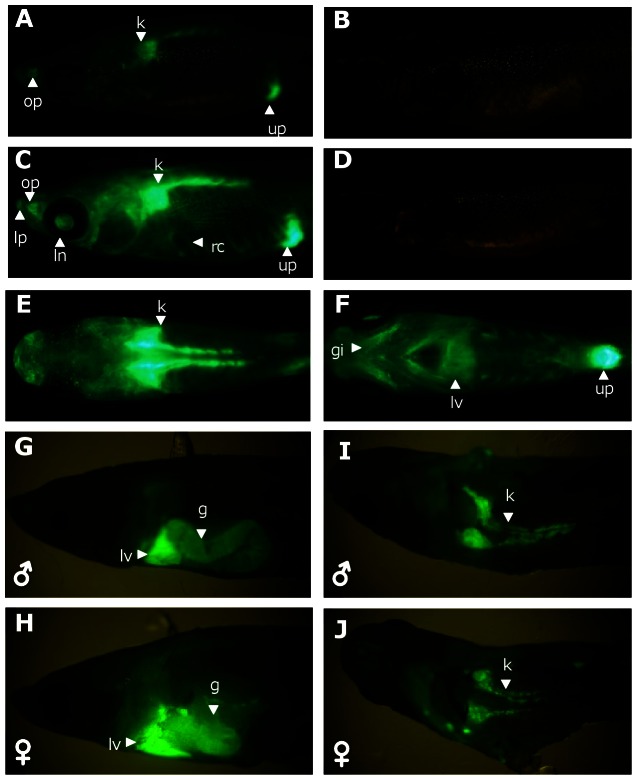
GFP induction of adult *Tg(cyp1a:gfp)* medaka by TCDD. (A, B) Lateral views of GFP induction of a representative male *Tg(cyp1a:gfp)* fish at 24 hrs after 2.5 nM TCDD exposure (A) in comparison with an untreated control fish (B). (C, D) Lateral views of GFP induction of a representative male *Tg(cyp1a:gfp)* fish at 72 hrs after 2.5 nM TCDD exposure.(C) in comparison with an untreated control fish (D) (E, F) Dorsal view (E) and ventral view (F) of the same fish from (C). *Tg(cyp1a:gfp)* fish in other TCDD concentrations (0.1 nM and 0.5 nM) displayed similar expression pattern and intensity to those shown here in (A, C, E, F). (G, H) GFP expression in the internal organs of a TCDD-treated male transgenic fish. (I, J) GFP expression in the internal organs of a TCDD-treated female transgenic fish. Lateral view for the liver and gut is shown in (G, I) and ventral view for the kidney after removal of internal digestive organs is shown in (H, J). Abbreviations: g, gut; gi, gills; k, kidney; ln, lens; lp, lips; lv, liver; op, olfactory pit; rc, ribcage; up, urinary pore.

To test whether GFP induction could be directly observed from live adult *Tg*(*cyp1a:gfp*) medaka, 6-month-old adult transgenic fish were treated with three different concentrations (0.1, 0.5 and 2.5 nM) of TCDD for 72 hours. After 24 hours of treatment, 2 male and 2 female *Tg*(*cyp1a:gfp*) fish from each of the three TCDD concentration groups were randomly picked and observed. In all the fish observed, GFP was expressed in olfactory pits as well as in the abdomen region corresponding to the kidney, liver and urinary pore positions (Figure 5A) while no GFP signal was observed in non-treated control (Figure 5B). After 72 hours of exposure, increasing GFP fluorescence was observed in the head, body trunk, skin and brightly in kidney, urinary pores, gills region, lens, lips and olfactory pit as well as rib cage in all fish from the three TCDD-treated groups (Figure 5C,E,F) and again there was no GFP signal observed in untreated control fish (Figure 5D). To confirm the GFP expressing organs, we dissected all the fish at the end of the 72-hour exposure in order to view its internal organs. In both TCDD-treated males (Figure 5G,H) and females (Figure 5I,J), the liver was found to have the most intensive GFP signal, next by the gut (Figure 5G,I). The GFP signal was also strong in the head kidney and kidney tubules (Figure 5H,J). Interestingly, the abdomen wall that was dissected out did not fluorescence, indicating that the earlier mentioned GFP signal in the rib cage was due to the strong signal from the internal organs which illuminated the rib cage. This observation indicate the feasibility to use live adult *Tg(cyp1a:gfp)* fish to develop an *in vivo* biomonitoring system for TCDD and related chemicals.

## Discussion

### Sensitivity of *Tg(cyp1a:gfp)* medaka

Previously we have generated several *Tg(cyp1a:gfp)* medaka for monitoring TCDD and other POPs [18] and the selection of *cyp1a* promoter is because *cyp1a* is the most reliable and robust responsive genes to TCDD [20], [21]. From multiple Tg(*cyp1a:gfp*) founders, here we isolated and characterized a highly sensitive transgenic line, where GFP expression was detected in the liver, kidney, gut and several other tissues/organs when treated with TCDD. In addition, this line has the highest sensitivity towards TCDD among the four *Tg(cyp1a:gfp)* families that we have maintained and compared. The LOEC of TCDD to evoke GFP expression is around 0.005 nM (Figure 1I) in the hemizygous *Tg(cyp1a:gfp)* 1–3 dph fry. The TCDD detection limit of the traditional EROD assay from cell culture is ranged from 2.4 pM to 1 nM [22], [23]. Thus, the LOEC we estimated based on our transgenic fish assay is quite comparable to the best EROD sensitivity reported. In comparison, some limitations exist in the EROD assay. Firstly, unknown chemicals in the sample could act as Cyp1a enzyme antagonists, potentiators or competitors, thus affecting the accuracy of the EROD assay [7], [24], [25]. Secondly, EROD assay from cell culture studies has shown to decrease at high concentrations of inducer, presumably due to inhibition of Cyp1a enzyme activity by the high concentration of inducer [23], [26], [27]. Thirdly, external parameters such as temperature, pH, etc. can also influence the outcome of EROD assay [7], [25], [28]. Transgenic fish assay can avoid these problems as it monitors TCDD more directly from the upstream transcriptional activity, which is controlled by TCDD-AhR interaction. Thus this transgenic model provide a better in vivo model for more direct monitoring of these environmental chemicals than EROD assays.

Compared to several cell-based transgenic assays using similar dioxin responsive elements linked luciferase reporter gene [22], [24], [29], [30], our transgenic fish assay is less sensitive as it has been reported in several cell-based assays have the detection limit from 0.8 pM to 1.0 pM of TCDD. The higher sensitivity of the transgenic cell cultures could be due to the direct exposure of cells by the toxicants while transgenic fish have additional physiological barrier against xenobiotics intake. However, our transgenic fish represent an authentic *in vivo* biological model and it is easy to perform by direct observation of easily scorable GFP fluorescence in live fry or adult fish. Moreover, the result is instant and the assay is economical compared to the EROD and transgenic cell-based assays. As fish naturally inhabit in the water environment, the transgenic medaka is an ideal model for developing into an online monitoring systems to report aquatic contamination by TCDD and similar pollutants.

### Specificity of *Tg(cyp1a:gfp)* medaka

We have further tested *Tg*(*cyp1a:gfp*) medaka fry with other known Cyp1a inducer such as 3-MC and BaP. Different from TCDD, BaP and 3-MC evoked GFP expression in the kidney, liver and gut only. TCDD apparently induced the highest and strongest reaction of *Tg(cyp1a:gfp)* among the three chemicals and at a concentration much lower than BaP and 3-MC, consistent with many previous reports that TCDD has the greatest affinity for AhR [20], [27], [31]. Perhaps, the concentrations of BAP and 3-MC used in this study may not be high enough to warrant GFP expression in other organs. Other chemicals such as BPA, lindane and mercury chloride, all of which are unknown to be AhR ligands, were tested on *Tg*(*cyp1a:gfp*) fry and basically there was no induced GFP expression observed even with their lethal dosage and longer exposure periods. Only weak GFP induction was observed in small percentage of fry in high concentrations of 4-nitrophenol. Thus, these observations demonstrated that *cyp1a* promoter based transgenic line is capable of responding specifically to AhR ligands such as TCDD, BAP and 3-MC. However, this transgenic line is unable to predict the exact chemical from its responses.

Although *cyp1a* transcription is primarily regulated by AhR pathway, *cyp1a* expression is also influenced by hormonal factors such as glucocorticoids, insulin and sex hormones [32]. These factors may directly and/or indirectly affect the *cyp1a* expression by crosstalking with other transcription factors. Furthermore, AhR pathway was suggested to be involved in other functions such as immunology and development in addition to the mediation of biological responses to chemicals and pollutants [33]. It has been reported that administration of AhR ligands, such as beta-naphthoflavone, modulates the induction of estrogen-receptor mediated downstream gene, suggesting that AhR pathway does crosstalk with estrogen receptor pathway [32], [33], [34], [35]. However, we observed no obvious difference between the GFP induction of transgenic adult male and female fish towards TCDD treatment, suggesting that the sex hormones did not significantly affect the transgenic responses towards TCDD.

### Spatial induction of GFP expression in *Tg(cyp1a:gfp)* medaka

A major advantage of *Tg(cyp1a:gfp)* medaka over cell-based transgenic models is the direct detection of tissue-distribution of induced GFP expression. Interestingly, induction of GFP expression was first observed in the liver, kidney and gut by TCDD (Figure 1I) as well by BAP and 3-MC (Figure 3D,E). *In situ* hybridization (Figure 2B) of TCDD-treated wild type fry demonstrated similar induction of endogenous *cyp1a* mRNAs in the liver, kidney and gut (Figure 2B). Consistent with our observation, strong immunohistostaining of Cyp1a has also been observed in the liver, kidney and gut lining after TCDD treatment in zebrafish as well as in other fish species [25], [36]. It is generally shown that the liver and kidney are major sites of xenobiotic metabolism and excretion, thus resulting in high level of Cyp1a activity. Due to dietary exposure, accumulation of xenobiotics could occur in the gut and induce Cyp1a [25]. However, in our exposure treatments, the fry were not fed yet but the gut was still a major organ for induced GFP expression. Thus, it is possible that gastrointestinal tract also acts as a major organ for detoxification. Interestingly, in 3-MC treatment, the percentage of fry with GFP induction in the gut was not as high as those in BAP and TCDD treatments, signifying differential organ affinity to different chemicals or differential distribution of different chemicals in different organs.

In addition to GFP induction in the kidney, liver and gut in Tg(*cyp1a:gfp*) fish, GFP was also induced in epithelial surface such as neuromast cells, olfactory pits, gills and skin. These have been confirmed by *in situ* hybridization (Figure 2E,H,K). Noticeably, neuromast cells, olfactory pits, gills and skins were organs that were in direct contact with toxicants in water; thus, non-enriched TCDD may directly interact with the AhR pathway while in the metabolic organs including the liver, kidney and gut, TCDD could be enriched and thus achieve higher response. It has also been reported from several fish studies that Cyp1a is present in olfactory pits, gills and skins in AhR-ligands treated fishes by immunostaining [25], [36]. To our knowledge, our observation that *cyp1a* gene is inducible by TCDD in neuromast cells is a novel finding.

Fewer organs showed *cyp1a* mRNA induction by *in situ* hybridization analysis than those displayed induced GFP expression in *Tg(cyp1a:gfp)* fish under the same TCDD chemical treatment. Organs that expressed GFP but not detected by in situ hybridization include blood vessels along the trunk of the body (Figure 2F), caudal fin (Figure 1F 4,G4,H4) and heart muscles. The difference between the two assays may reflect a detection sensitivity rather than ectopic GFP induction in the transgenic medaka. Consistent with our argument, it has been previously reported that Cyp1a induction is detected in vascular endothelia, myocardium and caudal fin of treated fish in whole mount and section immunostaining [25], [36], [37]. Nonetheless, GFP expression in the blood vessels, heart muscles and caudal fin was rather weak in comparison to the intense GFP signal in the major organs such as liver, kidney and gut (Figure 2C); thus, whole mount *in situ* hybridization is unable to detect the low level of expression.

We also noticed a trend that an increasing number of organs express GFP with increasing TCDD concentration. The order of these organs expressing GFP from low to high concentrations of TCDD is as follows: Kidney and liver>Gut>olfactory pits and caudal fin>gills>neuromast cells (Figure 1). Although the phenotypic expression was difficult to quantify with concentration, the order of organs that expressed GFP can be a rough indicator of relative dosage of TCDD.

### *Tg(cyp1a:gfp)* medaka for biomonitoring assays

We have tested *Tg(cyp1a:gfp)* medaka with TCDD at three different development stages: pre-hatching embryos, post-hatching fry and adult. In all the three stages, GFP was induced within 24 hours of TCDD exposure. However, the induced GFP expression in early embryos was relatively weak compared to the post-hatching fry and adults. This is likely due to both of the presence of thick chorion in pre-hatching embryos, which may affect the permeability of chemicals, and the weak AhR activity before the metabolic organs become mature. Consistent with this, we did observe stronger GFP expression induced by TCDD in the kidney, liver and gut at 3 dpf when these organs begin to develop (stage 19–26) [17]. Although strong GFP expression was observed in the kidney and olfactory pits in the adult transgenic fish after 24 hours of exposure, due to the presence of the thick body wall, GFP expression within abdomen area could not be sensitively observed unless dissected. This problem could be overcome by inserting the cyp1a:gfp construct in the see-through medaka strain [38], which remains transparent in both embryonic and adult stages, thus allowing fluorescence in internal organs to be observed directly without a need to sacrifice the animal. Currently, larvae stage remains the best stage for biomonitoring due to its complete and functional organs development and also the ease of detection in its transparent body at this stage.

Based on the data presented here, it is feasible to develop two biomonitoring systems using the *Tg(cyp1a:gfp)* medaka. One is to use the newly hatched fry as they offer maximal sensitivity to TCDD induction and the assay can be performed for at least 72 hours without feeding since the newly hatched fry can rely on their yolk reserve in the first few days after hatching. The assay can be used for large scale of chemical screening for xenobiotic activity because of the easy availability of a large number of fry. Another assay is based on the adult and it can be developed into both laboratory-based and on-site monitoring systems for both acute and chronic exposure. For on-site monitoring, medaka offer additional advantages as medaka fish are very hardy and can adapt in a broad salinity (up to 50% sea water) and a wide range of temperature from near 4°C to 40°C [15], [39]. Thus, *Tg(cyp1a:gfp)* transgenic medaka can be used in broad environmental conditions compared to most other fish species including the popular zebrafish model.

## Supporting Information

Figure S1 **Whole mount** ***in situ*** **hybridization with** ***cyp1a*** **sense probe in newly hatched fry in 0.1% DMSO vehicle solvent control (A) or 5 nM TCDD (B).**
(PDF)

Figure S2 **Weak GFP induction by** ***Tg(cyp1a:gfp)*** **fry by 4-nitrophenol.** (A) Lack of detectable GFP expression in a fry in the control (egg water) group. (B) Spotty GFP expression in the liver from a fry in the 4-nitrophenol treatment group. (C) Percentages of fry expressing GFP in the liver induced by 4-nitrophenol. Abbreviation: lv, liver.
(PDF)

Figure S3 **Transient and consitutive GFP expression in the mid-brain of** ***Tg(cyp1a:gfp)*** **embryos at 1 dpf.** (A) Brightfield view. (B) Fluorescent view. (C) Merged view. The GFP signal is diminished by 2 dpf. White arrowheads point to the positions of GFP expression and astrisks denote eyes.
(PDF)
