# Supplementary material for: GFP Transgenic Medaka (Oryzias latipes) under the Inducible cyp1a Promoter Provide a Sensitive and Convenient Biological Indicator for the Presence of TCDD and Other Persistent Organic Chemicals
Source: PLoS One. 2013 May 20;8(5):e64334. doi: 10.1371/journal.pone.0064334 (PMC3659123; doi:10.1371/journal.pone.0064334)
Supplement: Figure S1 — Whole mount in situ hybridization with cyp1a sense probe in newly hatched fry in 0.1% DMSO vehicle solvent control (A) or 5 nM TCDD (B). (PDF) [file pone.0064334.s001.pdf]

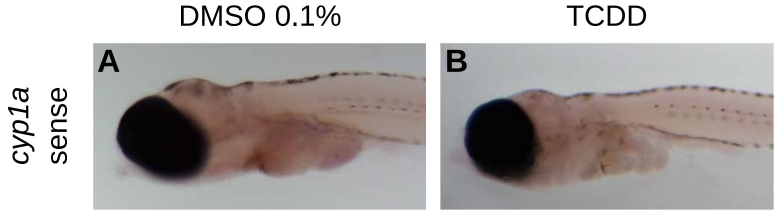

Figure S1. Whole mount *in situ* hybridization with *cyp1a* sense probe in newly hatched fry in 0.1% DMSO vehicle solvent control (A) or 5 nM TCDD (B).
