# Supplementary material for: GFP Transgenic Medaka (Oryzias latipes) under the Inducible cyp1a Promoter Provide a Sensitive and Convenient Biological Indicator for the Presence of TCDD and Other Persistent Organic Chemicals
Source: PLoS One. 2013 May 20;8(5):e64334. doi: 10.1371/journal.pone.0064334 (PMC3659123; doi:10.1371/journal.pone.0064334)
Supplement: Figure S2 — Weak GFP induction by Tg(cyp1a:gfp) fry by 4-nitrophenol. (A) Lack of detectable GFP expression in a fry in the control (egg water) group. (B) Spotty GFP expression in the liver from a fry in the 4-nitrophenol treatment group. (C) Percentages of fry expressing GFP in the liver induced by 4-nitrophenol. Abbreviation: lv, liver. (PDF) [file pone.0064334.s002.pdf]

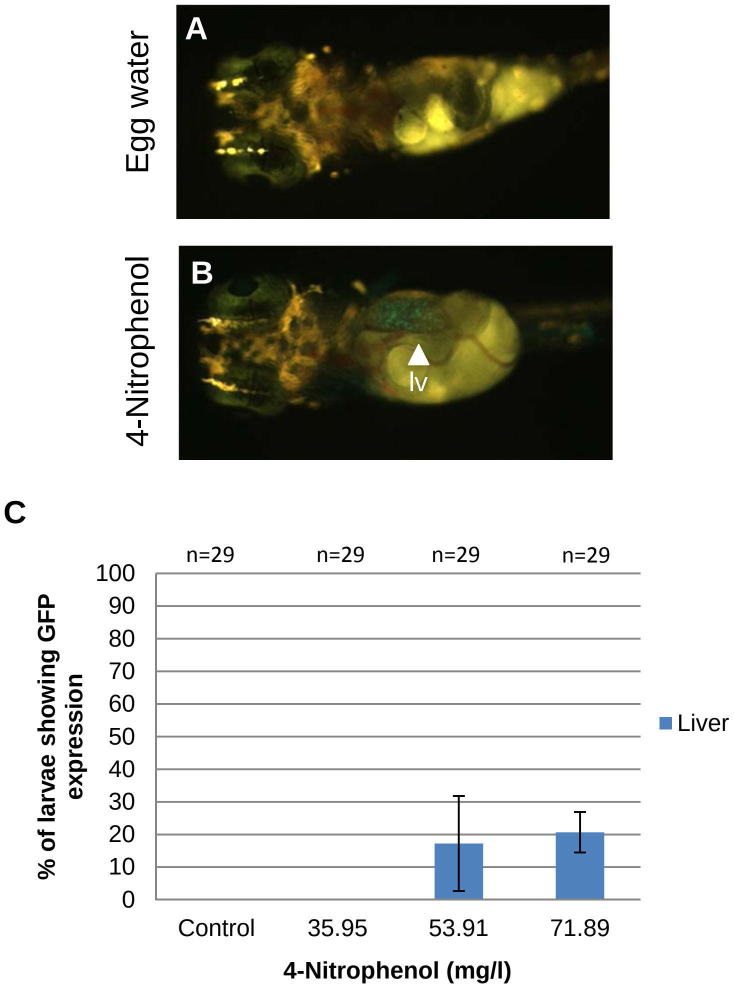


**Figure S2. Weak GFP induction by *Tg(cyp1a:gfp)* fry by 4-nitrophenol.** (A) Lack of detectable GFP expression in a fry in the control (egg water) group. (B) Spotty GFP expression in the liver from a fry in the 4-nitrophenol treatment group. (C) Percentages of fry expressing GFP in the liver induced by 4-nitrophenol. Abbreviation: lv, liver.
