# Supplementary material for: GFP Transgenic Medaka (Oryzias latipes) under the Inducible cyp1a Promoter Provide a Sensitive and Convenient Biological Indicator for the Presence of TCDD and Other Persistent Organic Chemicals
Source: PLoS One. 2013 May 20;8(5):e64334. doi: 10.1371/journal.pone.0064334 (PMC3659123; doi:10.1371/journal.pone.0064334)
Supplement: Figure S3 — Transient and consitutive GFP expression in the mid-brain of Tg(cyp1a:gfp) embryos at 1 dpf. (A) Brightfield view. (B) Fluorescent view. (C) Merged view. The GFP signal is diminished by 2 dpf. White arrowheads point to the positions of GFP expression and astrisks denote eyes. (PDF) [file pone.0064334.s003.pdf]

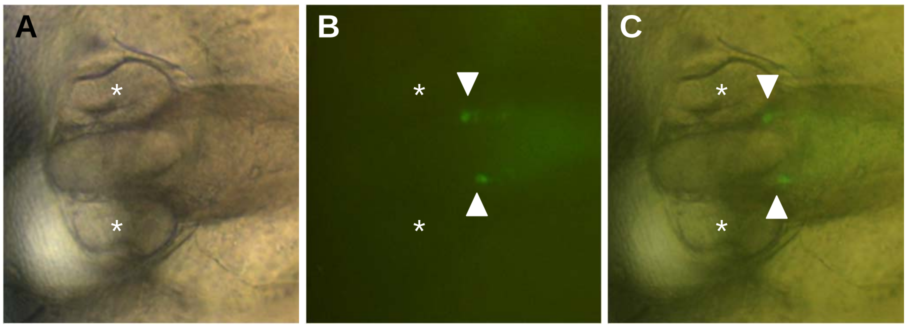

Figure S3. Transient and consitutive GFP expression in the mid-brain of *Tg(cyp1a:gfp)* embryos at 1 dpf. (A) Brightfield view. (B) Fluorescent view. (C) Merged view. The GFP signal is diminished by 2 dpf. White arrowheads point to the positions of GFP expression and astrisks denote eyes.
